# Modifiable lifestyle factors and 4.9-Year changes in phenotypic age in the Taiwan biobank

**DOI:** 10.3389/fragi.2026.1802176

**Published:** 2026-05-07

**Authors:** Wan-Yu Lin

**Affiliations:** 1 Institute of Health Data Analytics and Statistics, College of Public Health, National Taiwan University, Taipei, Taiwan; 2 Master of Public Health Program, College of Public Health, National Taiwan University, Taipei, Taiwan

**Keywords:** aging, biomarkers, DNA methylation, incense burning, oxidative stress

## Abstract

**Introduction:**

Phenotypic age (PhenoAge) is a composite biomarker that reflects biological aging by integrating indicators of immune, metabolic, liver, and kidney function.

**Methods:**

Using 4.9 years of follow-up data from 69,462 participants in the Taiwan Biobank, this study investigated lifestyle and environmental factors associated with changes in PhenoAge (ΔPhenoAge: follow-up minus baseline).

**Results:**

Partial correlation analysis was first applied to evaluate 43 lifestyle factors. Fifteen factors were positively correlated with ΔPhenoAge, with obesity-related indices showing the strongest correlations. Nine factors were inversely correlated, including employment status and dietary habits. These variables were further evaluated using best-subset regression to identify the most relevant factors. In multivariable analyses, ΔPhenoAge in men was associated with living alone, higher body fat percentage, and larger waist circumference. In women, ΔPhenoAge was associated with daily supplement use, eating supper within 1 h of bedtime, and higher body mass index. Exposure to incense burning was associated with higher ΔPhenoAge in both sexes. Conversely, reduced consumption of fried foods, greater variety of vegetables, lower meat intake, and staying at the same job throughout the career were associated with lower ΔPhenoAge.

**Discussion:**

These results suggest that several modifiable factors are associated with changes in biological aging, highlighting the importance of lifestyle for healthy aging.

## Introduction

Biological age, quantified using composite biomarker indices, can reflect an individual’s physiological condition. Among available approaches, phenotypic age has been shown to predict mortality and morbidity risk across diverse populations (Klemera and Doubal, 2006; Levine, 2013; Rutledge et al., 2022; Zhang et al., 2014). More recently, pathway-enrichment analyses have suggested that biological age estimation should incorporate indices across four major domains: immunity, metabolism, liver dysregulation, and kidney dysregulation (Ahadi et al., 2020). In line with this finding, Levine et al.’s “phenotypic age” (PhenoAge) aggregates biomarkers within these domains (Levine et al., 2018).

In 2018, Levine et al. developed the PhenoAge metric to assess human biological aging (Levine et al., 2018). Using clinical data from the third National Health and Nutrition Examination Survey (NHANES III), the hazard of mortality was regressed on chronological age and 42 clinical biomarkers. Based on 10-fold cross-validation, nine biomarkers, along with chronological age, were identified as the most informative predictors of mortality risk. These variables were combined using weighted linear coefficients and subsequently transformed through a Gompertz mortality model to estimate human biological age. PhenoAge has been shown to effectively capture morbidity and mortality risk across diverse populations (Liu et al., 2019).

Among the nine biomarkers aggregated by PhenoAge, four were related to immune or hematologic function: lymphocyte percentage, **mean corpuscular volume**, red cell distribution width, and **white blood cell count**. Two biomarkers reflected liver function: **albumin** and alkaline phosphatase. In addition, three other biomarkers were used to represent kidney function (**creatinine**), metabolic condition (**fasting serum glucose**), and systemic inflammation (C-reactive protein). Of these nine biomarkers, the five highlighted in bold were available in the Taiwan Biobank (TWB), while the remaining four were not measured. Importantly, the five available biomarkers covered the four key domains—immunity, metabolism, liver function, and kidney function—considered critical for biological age estimation (Ahadi et al., 2020).

Because the TWB provided six markers—chronological age and the five biomarkers—Lin evaluated the performance of a “6markerPhenoAge” for estimating PhenoAge (Lin, 2022). First, the “6markerPhenoAge” was calculated for 9,598 participants from NHANES III. Sex-stratified scatter plots demonstrated a strong linear relationship between “6markerPhenoAge” and PhenoAge. Pearson’s correlation coefficients were 0.960 among 4,483 men and 0.936 among 5,115 women. Based on the strong linear associations observed in the scatter plots and the high correlation coefficients, a linear formula was subsequently developed to estimate PhenoAge using “6markerPhenoAge” (Lin, 2022).

Genetic influences on PhenoAge appear to be relatively modest. SNP-based heritability estimates for PhenoAge were 14.45% (standard error [SE] = 0.95%) in the United Kingdom Biobank (Kuo et al., 2021) and 14.03% (SE = 1.49%) in the TWB (Lin, 2022). These findings suggest that non-genetic factors may play an important role in shaping PhenoAge. Leveraging 4.9 years of follow-up data from 69,462 TWB participants, the present study systematically evaluated multiple lifestyle factors to identify those associated with changes in PhenoAge. Understanding these associations may provide insight into modifiable risk factors for biological aging.

## Materials and methods

### The Taiwan biobank data

From 2008 to 2025, the TWB recruited approximately 200,000 community-based volunteers from Taiwan’s residents, among whom ∼87,000 completed the first follow-up survey (Wei et al., 2021). Baseline was defined as the initial assessment at enrollment (between 2008 and 2022), and follow-up as the first follow-up visit (between 2011 and 2025). The mean follow-up duration was 4.9 years (range: 1.6–15.9 years). Participants were aged above 20 years without a history of cancer diagnosis. Written informed consent was obtained from all participants. Each individual underwent standardized physical health examinations conducted by trained health professionals, provided blood and urine samples, and completed a structured face-to-face lifestyle questionnaire.

The urine and blood samples were analyzed by laboratories certified to ISO standards and accredited by the College of American Pathologists (CAP). The TWB questionnaire included personal information, dietary habits, and environmental exposures. Epidemiologists designed the questionnaire, and several workgroups evaluated its clarity and logical flow. Moreover, the TWB performed a preliminary study to validate the questionnaire. The reliability of the TWB questionnaire was evaluated by comparing participants’ responses at baseline and follow-up surveys (Feng et al., 2022). The 43 lifestyle factors were described in Tables 1, 2 and the Supplementary Material.

**TABLE 1:** Continuous factors.

Lifestyle factors	Baseline		Follow-up	
​	Men (N = 25,375)	Women (N = 44,087)	Men (N = 25,375)	Women (N = 44,087)
PhenoAge (years)	44.97 ± 12.68	42.01 ± 11.07	49.72 ± 12.81	46.62 ± 11.21
Chronological age (years)	50.15 ± 11.28	49.87 ± 10.41	55.06 ± 11.26	54.75 ± 10.38
Body mass index (kg/m^2^)	25.26 ± 3.50	23.45 ± 3.66	25.45 ± 3.59	23.72 ± 3.77
Body fat percentage (%)	22.82 ± 5.32	31.75 ± 6.29	22.94 ± 5.32	32.43 ± 6.36
Waist circumference (cm)	87.52 ± 9.20	80.24 ± 9.50	88.54 ± 9.47	81.20 ± 9.74
Hip circumference (cm)	97.36 ± 6.65	94.96 ± 7.06	97.18 ± 6.76	95.16 ± 7.27
Waist-to-hip ratio	0.90 ± 0.06	0.84 ± 0.07	0.91 ± 0.06	0.85 ± 0.07
Diet1^a^	​	​	2.56 ± 1.61	3.28 ± 1.62
Diet2^a^	​	​	2.82 ± 1.25	2.98 ± 1.32
Diet3^a^	​	​	2.49 ± 0.98	2.63 ± 1.03
Diet4^a^	​	​	3.73 ± 1.25	4.13 ± 1.08
Diet5^a^	​	​	3.87 ± 0.91	4.05 ± 0.85
Diet6^a^	​	​	4.40 ± 0.95	4.42 ± 0.93
Diet7^a^	​	​	3.69 ± 1.21	3.87 ± 1.14
Diet8^a^	​	​	4.29 ± 0.73	4.37 ± 0.68
Diet9^a^	​	​	3.36 ± 1.46	3.21 ± 1.44
Diet10^a^	​	​	2.79 ± 1.43	2.63 ± 1.45
Diet11^a^	​	​	3.54 ± 1.61	3.34 ± 1.64
Diet12^a^	​	​	3.45 ± 1.63	3.58 ± 1.61
Diet13^a^	​	​	3.33 ± 1.47	3.21 ± 1.51
Diet14^a^	​	​	3.22 ± 1.56	2.79 ± 1.59
Diet15^a^	​	​	3.35 ± 1.42	3.02 ± 1.44
Diet16^a^	​	​	1.63 ± 0.94	1.68 ± 0.99
Diet17^a^	​	​	3.64 ± 1.57	3.31 ± 1.67
Educational attainment score^b^	​	​	5.75 ± 0.87	5.45 ± 0.96

^a^ The 17 diet-related questions are listed in Supplementary Table S1. Because lifestyle factors were more comprehensively surveyed at follow-up than at baseline, the lifestyle factors used in this study were primarily collected at follow-up.

^b^ Educational attainment score: Educational attainment was coded as an integer ranging from 1 to 7: 1 (illiterate), 2 (no formal education but literate), 3 (primary school graduate), 4 (junior high school graduate), 5 (senior high school graduate), 6 (college graduate), and 7 (Master’s or higher degree).

**TABLE 2:** Ordinal and dichotomous lifestyle factors.

Lifestyle factors	Men	Women
Sample size	25,375	44,087
Ordinal lifestyle factors		
Cooking^a^	​	​
Cooking = 1: did not cook by yourself	18,111 (71.4%)	9,582 (21.7%)
Cooking = 2: Sometimes cook by yourself	163 (0.6%)	479 (1.1%)
Cooking = 3: Regularly cook by yourself	7,101 (28.0%)	34,026 (77.2%)
Nut^b^	​	​
Nut = 1: no betel nut chewing	24,527 (96.7%)	44,046 (99.9%)
Nut = 2: Betel nut chewing sometimes	615 (2.4%)	34 (<0.1%)
Nut = 3: Betel nut chewing almost daily	233 (0.9%)	7 (<0.1%)
Supplement ^c^	​	​
Supplement = 1: Do not take supplements	11,458 (45.2%)	14,688 (33.3%)
Supplement = 2: Sometimes take supplements	5,128 (20.2%)	12,454 (28.2%)
Supplement = 3: Regularly take supplements	8,789 (34.6%)	16,945 (38.4%)
Dichotomous lifestyle factors		
Living alone	1,680 (6.6%)	4,343 (9.9%)
Job ^d^	17,181 (67.7%)	25,624 (58.1%)
JobSame ^e^	2,307 (9.1%)	3,741 (8.5%)
Medication ^f^	153 (0.6%)	319 (0.7%)
Coffee ^g^	12,002 (47.3%)	20,825 (47.2%)
Tea ^h^	7,589 (29.9%)	8,330 (18.9%)
Drinking ^i^	3,853 (15.2%)	1,091 (2.5%)
Smoking ^j^	4,204 (16.6%)	990 (2.2%)
Smk2nd ^k^	3,189 (12.6%)	3,483 (7.9%)
Incense ^l^	5,861 (23.1%)	11,597 (26.3%)
Vege ^m^	1,905 (7.5%)	4,463 (10.1%)
Supper ^n^	9,712 (38.3%)	13,562 (30.8%)
SPOYesYes ^o^	8,166 (32.2%)	12,703 (28.8%)
SPOYesNo ^p^	2,860 (11.3%)	4,856 (11.0%)
SPONoYes ^q^	3,335 (13.1%)	6,043 (13.7%)
SPONoNo ^r^	11,002 (43.4%)	20,456 (46.4%)

^a^ Cooking = 1: have not cooked meals by yourselves for more than 6 months; Cooking = 2: cooked meals by yourselves sometimes; Cooking = 3: have cooked by yourselves for over 6 months.

^b^ Nut = 1: no betel nut chewing; Nut = 2: betel nut chewing sometimes; Nut = 3: betel nut chewing almost daily.

^c^ Supplement = 1: do not take vitamins, minerals, or supplements in the past month before joining the TWB; Supplement = 2: sometimes (not regularly) take vitamins, minerals, or supplements in the past month before joining the TWB; Supplement = 3: regularly take vitamins, minerals, or supplements in the past month before joining the TWB.

^d^ Job: Currently having a job.

^e^ JobSame: Stay at the same job throughout the career.

^f^ Medication: taking cough syrup, sedatives, or pain relievers at least once a week within the 6 months before joining the TWB.

^g^ Coffee: coffee drinking at least three times a week.

^h^ Tea: consuming tea (containing tea leaves, excluding herbal tea) at least once daily within 6 months before joining the TWB.

^i^ Drinking: having a weekly intake of more than 150 mL of alcoholic beverages for at least 6 months when joining the TWB.

^j^ Smoking: having smoked cigarettes for at least 6 months when joining the TWB.

^k^ Smk2nd: being exposed to an environment with second-hand smoke (someone smoking nearby) for at least five minutes in the past 6 months.

^l^ Incense: being exposed to incense burning (e.g., during worship or the use of incense powder or rings), mosquito coils (traditional, liquid electric, or electric mosquito repellent), or fragrances (such as essential oils, aromatherapy, air fresheners, sprays, or scented candles) for at least five minutes in the past year before joining the TWB.

^m^ Vege: a vegetarian diet for at least 6 months before joining the TWB.

^n^ Supper: eating supper within an hour before bedtime (including milk and wine).

^o^ SPOYesYes: regularly performing exercise (performing exercise lasting for 30 min, three times per week) at baseline and follow-up.

^p^ SPOYesNo: regularly performing exercise (performing exercise lasting for 30 min, three times per week) at baseline but not at follow-up.

^q^ SPONoYes: regularly performing exercise (performing exercise lasting for 30 min, three times per week) at follow-up but not at baseline.

^r^ SPONoNo: neither regularly performing exercise (performing exercise lasting for 30 min, three times per week) at baseline nor at follow-up.

PhenoAge was calculated using the sex-specific equations proposed by Lin (Lin, 2022), in which PhenoAge is a linear transformation of the 6-marker PhenoAge. Specifically, PhenoAge was derived as 45.846752 + 1.068403 × 6markerPhenoAge for men and 46.527973 + 1.023272 × 6markerPhenoAge for women. The 6-marker PhenoAge was calculated using a mortality-score formulation based on the method of Levine et al. (2018), incorporating six biomarkers available in TWB: mean corpuscular volume, white blood cell count, albumin, creatinine, fasting serum glucose, and chronological age. Detailed coefficients and transformation functions were adopted directly from Levine et al. (2018).

### Description of diet and lifestyle factors

Lifestyle factors were primarily assessed using questionnaire data from the follow-up visit, as more comprehensive information was collected there than at baseline. Personal characteristics included living alone (yes/no), education level (1-7, ranging from illiterate to Master’s degree or higher), current employment status (yes/no), and job stability (defined as whether a participant remained in the same job throughout his/her career).

Five obesity-related metrics included body mass index (BMI), body fat percentage (BFP), waist circumference (WC), hip circumference (HC), and waist-to-hip ratio (WHR). According to the American Heart Association (AHA), these measures are considered modifiable risk factors (Lloyd-Jones et al., 2010).

Dietary habits were assessed using 17 questionnaire items (Diet1-Diet17; Supplementary Table S1), each describing a specific dietary behavior. Responses were recorded on a 5-point scale ranging from “Always” (1) to “Never” (5). Higher scores generally indicated healthier behaviors for Diet1-Diet8, whereas lower scores indicated healthier behaviors for Diet9-Diet17.

Regular exercise was defined as engaging in physical activity lasting at least 30 min, three times per week. Because regular exercise was surveyed at both baseline and follow-up, four indicator variables were defined to capture changes over time: consistent activity (SPOYesYes), baseline-only activity (SPOYesNo), follow-up-only activity (SPONoYes), and consistent inactivity (SPONoNo).

Environmental exposures included smoking status (≥6 months), second-hand smoke exposure (≥5 min within the past 6 months), and exposure to incense or related airborne substances (≥5 min within the past year). Additional lifestyle-related factors covered alcohol consumption, coffee and tea intake, vegetarian diet, late-night eating, and medication use, all defined using binary indicators based on frequency criteria. Three ordinal variables were also included: cooking frequency, betel nut chewing, and supplement use, each categorized into three levels reflecting increasing frequency. Detailed definitions of all factors are provided in Table 2.

### Statistical analysis

The primary objective of this study was to evaluate within-individual changes in phenotypic aging in relation to lifestyle factors. Therefore, ΔPhenoAge (PhenoAge at follow-up minus PhenoAge at baseline) was used to capture longitudinal change in biological aging within the same individual. In contrast, phenotypic age acceleration (= PhenoAge minus chronological age) reflects the cross-sectional deviation of PhenoAge from the expected age at a given time point rather than a temporal change. Given the focus on longitudinal change, ΔPhenoAge was used to address the study’s objective.

The analysis was conducted in two stages. First, partial correlation analyses (Pearson) were performed as an exploratory step to screen for potential associations between individual lifestyle factors and ΔPhenoAge, adjusting for sex and the follow-up time (ΔAge, chronological age at follow-up minus chronological age at baseline). These two variables were selected as essential adjustments to account for sex differences and between-individual variation in observation duration. Chronological age (in years) was calculated as the difference between the survey date (when urine, blood, and lifestyle data were collected) and the date of birth, divided by 365.25, with decimal values retained. Partial correlations were computed using the “pcor.test” function in the “ppcor” package in R (Kim, 2015).

Second, to identify the combination of lifestyle factors associated with ΔPhenoAge, lifestyle factors correlated with △PhenoAge at p < 0.05 were subsequently analyzed using best-subset analysis. Multivariable linear regression models were constructed separately for men and women; therefore, sex was not included as a covariate. The model with the smallest Akaike information criterion (AIC) was selected as the best model for △PhenoAge, while the follow-up time (ΔAge) was consistently retained in the final models. With the smallest AIC, the best model struck the optimal balance between model fit and parsimony (i.e., using fewer lifestyle factors). The “bestglm” package (https://cran.r-project.org/web/packages/bestglm/index.html) was used for the best-subset analysis. All statistical analyses were conducted in R (version 4.4.3). This two-stage approach enabled the identification of relevant variables and the evaluation of their joint associations with ΔPhenoAge.

## Results

### The lifestyle factors

**FIGURE 1:**
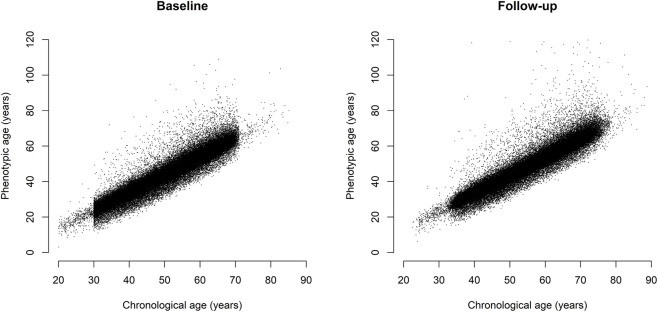
Relationship between phenotypic age and chronological age at baseline and follow-up. Scatter plots show phenotypic age (*y*-axis) versus chronological age (*x*-axis) at baseline (left) and follow-up (right). Each point represents one participant. The apparent vertical cutoff at age 30 in the baseline plot reflects the recruitment criteria of the Taiwan Biobank: individuals aged 30–70 years were enrolled prior to May 2014, whereas younger individuals (≥20 years) were included thereafter.

Figure 1 presents the relationship between phenotypic age and chronological age at baseline and follow-up. In addition, the trajectories of phenotypic age were illustrated for a randomly selected subset of participants (200 men and 200 women) in Supplementary Figure S1, as displaying all individuals would reduce the clarity of the visualization. Table 1 presents the means and standard deviations (SD) for continuous factors. A total of 69,462 participants had complete data for all 43 lifestyle factors, comprising 25,375 (36.5%) men and 44,087 (63.5%) women. The mean time between the baseline and follow-up was 4.91 years (SD = 1.51) for men and 4.88 years (SD = 1.53) for women. The mean change in PhenoAge was 4.75 years (SD = 4.01) for men and 4.61 years (SD = 3.50) for women. From baseline to follow-up, most people had an increase in PhenoAge. However, there were 7.8% of men and 6.7% of women had a negative △PhenoAge, highlighting that biological age can potentially be reversed to some extent. Except for men’s HC, all five obesity indices increased from baseline to follow-up.

As mentioned in the Supplementary Material, high points are usually considered better than low points for “Diet1” to “Diet 8”, whereas low points are generally healthier than high points for “Diet9” to “Diet17” (Supplementary Table S1). Compared with men, women had higher scores in “Diet1” to “Diet 8” and usually had lower scores in “Diet9” to “Diet17”. This survey showed that women generally developed healthier dietary habits than men. This result is consistent with that from a large Italian population sample: there is a gender difference in food preferences, and women tend to choose healthier foods (Feraco et al., 2024).

Table 2 shows the ordinal and dichotomous lifestyle factors. While most women (77.2%) cooked regularly on their own, most men (71.4%) did not. Very few people chewed betel nuts, especially women. Approximately 34.6% of men and 38.4% of women regularly take supplements, such as vitamins or minerals. More women (9.9%) lived alone than men (6.6%). A higher percentage of men (67.7%) held a job during the TWB than women (58.1%). A total of 9.1% of men and 8.5% of women stayed at the same job throughout their careers. Very few men (0.6%) and women (0.7%) used cough syrup, sedatives, or pain relievers weekly in the 6 months before joining the TWB. More individuals consumed coffee (47.3% of men and 47.2% of women) than tea (29.9% of men and 18.9% of women). More men drank alcohol and smoked than women (drinking: 15.2% vs. 2.5%; smoking: 16.6% vs. 2.2%). Furthermore, more men were exposed to second-hand smoke than women (12.6% vs. 7.9%). More women (26.3%) were exposed to incense burning (e.g., during worship or the use of incense powder or rings for at least five minutes within a year) than men (23.1%). More women (10.1%) followed a vegetarian diet than men (7.5%). More men (38.3%) ate supper within an hour before bedtime (including milk and wine) than women (30.8%).

When discussing physical exercise, men/women were categorized into four groups. Consistent across the two sexes, the largest group was “SPONoNo” (43.4% of men and 46.4% of women): no “regular exercise” (30 min, three times per week) at baseline or follow-up. The second-largest group was “SPOYesYes” (32.2% of men and 28.8% of women), who regularly engaged in exercise at both baseline and follow-up. Then there was “SPONoYes” (13.1% of men and 13.7% of women): regularly performing exercise at follow-up but not at baseline. The smallest group was “SPOYesNo” (11.3% of men and 11.0% of women), who regularly performed exercise at baseline but not at follow-up.

### Partial correlation coefficients

**FIGURE 2:**
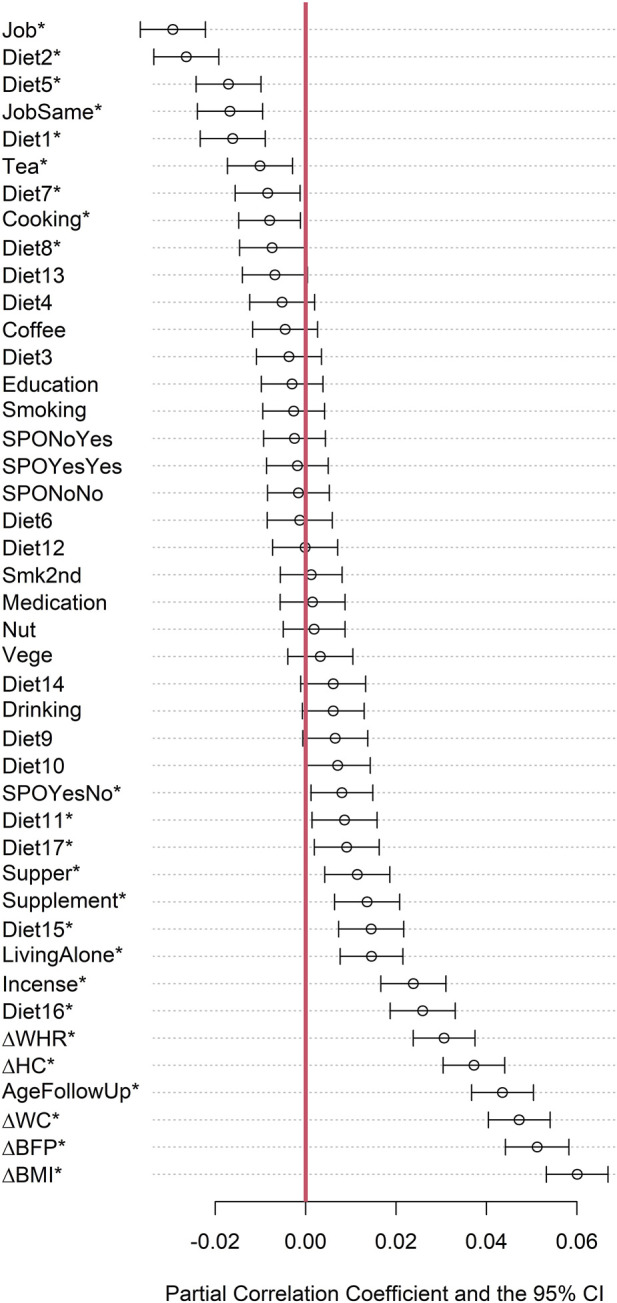
Partial correlation coefficients and the 95% confidence intervals of the 43 factors. The descriptions for each lifestyle factor can be found in Tables 1–3 or Supplementary Table S1 (Diet1, … , Diet17). A star (*) indicates a p-value <0.05 for the testing hypothesis H_0_: partial correlation coefficient = 0 vs. H_1_: partial correlation coefficient ≠ 0. Factors are sorted by the partial correlation coefficients.

Figure 2 presents the partial correlation coefficients and the 95% confidence intervals (CIs) between each of the lifestyle factors and the change in PhenoAge (i.e., △PhenoAge, PhenoAge at follow-up minus PhenoAge at baseline), while adjusting for sex and the follow-up time (i.e., △Age, chronological age at follow-up minus chronological age at baseline). Chronological age (in years) was calculated as the difference between the survey date (when urine and blood samples, and lifestyle data were collected) and the date of birth, divided by 365.25; decimal values were retained.

Fifteen factors were positively correlated with △PhenoAge (p < 0.05), including △BMI (i.e., BMI at follow-up minus BMI at baseline), △BFP, △WC, the chronological age at follow-up, △HC, △WHR, Diet16 (Do you eat at least two kinds of vegetables a day? 1: Always; …; 5: Never), Incense (=1 if an individual has been exposed to incense burning for at least five minutes in the past year, and 0 otherwise), living alone (=1 if an individual lives alone, and 0 otherwise), Diet15 (Would you choose a vegetarian and light diet in certain meals to reduce the intake of higher-fat foods such as meat or fat? 1: Always; …; 5: Never), Supplement (Supplement = 1: do not take vitamins, minerals, or supplements in the past month; Supplement = 2: sometimes take supplements; Supplement = 3: regularly take supplements), Supper (=1 if an individual eats supper within an hour before bedtime, and 0 otherwise), Diet17 (Do you intentionally eat less when having meat? 1: Always; …; 5: Never), Diet11 (If a food product has a low-fat option, would you choose it instead of the regular product? 1: Always; …; 5: Never), and SPOYesNo (=1 if performing exercise at baseline but not at follow-up, and 0 otherwise).

By contrast, nine factors were negatively correlated with △PhenoAge (p < 0.05), including Job (=1 if an individual currently has a job, and 0 otherwise), Diet2 (When you eat fish or meat, do you prefer to cook it with oil, such as frying? 1: Always; …; 5: Never), Diet5 (When you eat soy foods, do you prefer them deep-fried? 1: Always; …; 5: Never), JobSame (=1, if an individual stays at the same job throughout the career, and 0 otherwise), Diet1 (When you eat meat, do you eat it with fat, suet, or skin? 1: Always; …; 5: Never), Tea (=1, if an individual consumes tea daily, and 0 otherwise), Diet7 (When you have meals, do you add additional salt, soy sauce, chili sauce, or any other seasoning? 1: Always; …; 5: Never), Cooking (Cooking = 1: an individual does not cook by himself/herself for more than 6 months; Cooking = 2: sometimes cook; Cooking = 3: regularly cook), and Diet8 (Are you used to having pickles, fermented tofu, and fermented soybeans as side dishes in a meal? 1: Always; …; 5: Never).

Taken together, the five obesity indices were the most pronounced risk factors for PhenoAge acceleration. The chronological age at follow-up was positively correlated with PhenoAge acceleration, indicating that older people presented a faster aging rate. The baseline chronological age was not included as an evaluated factor because it could be determined solely from △Age and the chronological age at follow-up. Some diet-related questions were correlated with PhenoAge acceleration. As mentioned above, by common sense, higher points were generally considered healthier than lower points for Diet1 to Diet8, whereas the opposite was true for Diet9 to Diet17. Therefore, it was reasonable that Diet2, Diet5, Diet1, Diet7, and Diet8 were negatively correlated with △PhenoAge (p < 0.05), while Diet16, Diet15, Diet17, and Diet11 were positively correlated with △PhenoAge (p < 0.05).

### Best-subset analysis results

A total of 24 (=15 + 9) factors were significantly correlated with △PhenoAge (p < 0.05). These 24 factors and △Age (chronological age at follow-up minus chronological age at baseline) were further analyzed using the best-subset analysis to explain △PhenoAge. △Age is a recognized predictor because the time gap between baseline and follow-up varied for different individuals.

The best-subset analysis was performed for 25,375 men and 44,087 women, respectively. The largest variance inflation factors (VIFs) in both models were below 2, indicating no multicollinearity. Table 3 shows that the critical obesity indices for men’s PhenoAge acceleration were △BFP (p < 0.001) and △WC (p < 0.001). By contrast, △BMI (p < 0.001) was crucial for women’s PhenoAge acceleration. Living alone was associated with a 0.4032-year increase in men’s △PhenoAge (p < 0.001), but was not associated with women’s △PhenoAge. Diet2 (When you eat fish or meat, do you prefer to cook it with oil, such as frying? 1: Always; …; 5: Never) and Diet5 (When you eat soy foods, do you prefer them deep-fried, such as fried tofu? 1: Always; …; 5: Never) were inversely associated with both men’s and women’s △PhenoAge. The regression coefficients (Betas) listed in Table 3 need to be explained in terms of the units of the predictors. For example, men “always” cooking soy foods in a deep-fry way (coded as 1 in Diet5) were associated with a 0.3684 (=0.0921 × 4; p < 0.001) year increase in △PhenoAge compared with men “never” cooking soy foods in a deep-fry way (coded as 5 in Diet5).

**TABLE 3:** Results of the best-subset analysis.

Predictor (unit)	Men Beta (standard error)	Women Beta (standard error)
△Age ^a^ (in years)	1.2535 (0.0146) ***	1.1960 (0.0092) ***
Chronological age at follow-up	0.0155 (0.0024) ***	0.0236 (0.0015) ***
△BMI ^b^ (in kg/m^2^)	---	0.1478 (0.0092) ***
△BFP ^c^ (in %)	0.0379 (0.0077) ***	---
△WC ^d^ (in cm)	0.0174 (0.0047) ***	---
Living alone (yes vs. no)	0.4032 (0.0884) ***	---
Diet2 (1–5 points) ^e^	−0.0672 (0.0177) ***	−0.0683 (0.0108) ***
Diet5 (1–5 points) ^f^	−0.0921 (0.0251) ***	−0.0502 (0.0170) **
Diet16 (1–5 points) ^g^	0.0941 (0.0236) ***	0.0664 (0.0143) ***
Diet17 (1–5 points) ^h^	0.0321 (0.0142) *	0.0220 (0.0086) *
Cooking (1, 2, 3) ^i^	0.0576 (0.0245) *	−0.0566 (0.0180) **
Incense (yes vs. no) ^j^	0.2747 (0.0524) ***	0.1633 (0.0322) ***
Job ^k^	−0.1185 (0.0564) *	---
JobSame (yes vs. no) ^l^	−0.3710 (0.0758) ***	−0.0981 (0.0500) *
Tea (yes vs. no)	−0.1053 (0.0476) *	---
Supper ^m^	---	0.0700 (0.0307) *
Supplement (1, 2, 3) ^n^	---	0.0462 (0.0165) **
Adjusted R-square	24.59%	29.90%
The largest variance inflation factor (VIF) in model ^o^	1.60	1.25

Statistical significance is indicated by *, **, and ***, corresponding to p-values less than 0.05, 0.01, and 0.001, respectively.

^a^ △Age: chronological age at follow-up minus chronological age at baseline.

^b^ △BMI: body mass index at follow-up minus body mass index at baseline.

^c^ △BFP: body fat percentage at follow-up minus body fat percentage at baseline.

^d^ △WC: waist circumference at follow-up minus waist circumference at baseline.

^e^ Diet2: When you eat fish or meat, do you prefer to cook it with oil (such as by frying, deep-frying, braising, or steaming fish and topping it with oil)? 1: Always; 2: Most of the time; 3: Half of the time; 4: Seldom; 5: Never.

^f^ Diet5: When you eat soy foods, do you prefer them deep-fried (such as fried tofu, stinky tofu, or tofu skin)? 1: Always; 2: Most of the time; 3: Half of the time; 4: Seldom; 5: Never.

^g^ Diet16: Do you eat at least two kinds of vegetables a day? 1: Always; 2: Most of the time; 3: Half of the time; 4: Seldom; 5: Never.

^h^ Diet17: Do you intentionally eat less when having meat? 1: Always; 2: Most of the time; 3: Half of the time; 4: Seldom; 5: Never.

^i^ Cooking = 1: have not cooked meals by yourselves for more than 6 months; Cooking = 2: have cooked meals by yourselves sometimes; Cooking = 3: have regularly cooked by yourselves for over 6 months.

^j^ Incense: being exposed to incense burning (e.g., during worship or the use of incense powder or rings), mosquito coils (traditional, liquid electric, or electric mosquito repellent), or fragrances (such as essential oils, aromatherapy, air fresheners, sprays, or scented candles) for at least five minutes within the past year before joining the TWB.

^k^ Job: Currently having a job.

^l^ JobSame: Stay at the same job throughout the career.

^m^ Supper: eating supper within an hour before bedtime (including milk and wine).

^n^ Supplement = 1: do not take vitamins, minerals, or supplements in the past month before joining the TWB; Supplement = 2: sometimes (not regularly) take vitamins, minerals, or supplements in the past month before joining the TWB; Supplement = 3: routinely take vitamins, minerals, or supplements in the past month before joining the TWB.

^o^ The largest variance inflation factors (VIFs) in both models were below 2. No multicollinearity was found.

Diet16 (Do you eat at least two kinds of vegetables a day? 1: Always; …; 5: Never) and Diet17 (Do you intentionally eat less when having meat? 1: Always; …; 5: Never) were positively associated with men’s and women’s △PhenoAge. Men “never” eating at least two kinds of vegetables daily (coded as 5 in Diet16) were associated with a 0.3764 (=0.0941 × 4; p < 0.001) year increase in △PhenoAge compared with men “always” eating at least two kinds of vegetables daily (coded as 1 in Diet16). The association directions of these diet-related questions were consistent with those in partial correlation analysis (Figure 2).

A year increase in the chronological age at follow-up was associated with a 0.0155 (standard error [SE] = 0.0024; p < 0.001) year increase in men’s △PhenoAge and a 0.0236 (SE = 0.0015; p < 0.001) year increase in women’s △PhenoAge. This result indicated that older individuals showed greater increases in △PhenoAge. “Cooking by yourself” was associated with men’s PhenoAge acceleration (p < 0.05), but was associated with women’s PhenoAge deceleration (p < 0.01). This sex-specific association may be interpreted in the context of traditional gender roles in Taiwanese society. Traditionally, Taiwanese women handle more routine family duties, such as cooking (Iida, 2023). This phenomenon is also evident in TWB, where 71.4% of men reported not cooking, while 77.2% of women reported cooking regularly on their own (Table 2). For men, “cooking by yourself” may reflect “living alone”, which is a risk factor for men’s PhenoAge acceleration.

Consistent for both sexes, “exposure to incense burning” was a risk factor for men’s and women’s PhenoAge acceleration (p < 0.001). By contrast, “staying at the same job throughout the career” was associated with a 0.3710-year decrease in men’s △PhenoAge (p < 0.001) and a 0.0981-year decrease in women’s △PhenoAge (p < 0.05). “Tea consumption” was associated with men’s PhenoAge deceleration (p < 0.05). Furthermore, “eating supper within an hour before bedtime” (p < 0.05) and “regularly taking supplements like vitamins or minerals” (p < 0.01) were associated with women’s PhenoAge acceleration.

Summary of main findings. Overall, most individuals showed an increase in PhenoAge over time, while a small proportion showed a decrease, suggesting that biological aging may be modifiable. Among the 43 lifestyle factors examined, changes in obesity-related indices (e.g., BMI, body fat percentage, and waist circumference) showed the strongest and most consistent associations with ΔPhenoAge. Several dietary and lifestyle factors, including vegetable intake, cooking methods, supper habits, and supplement use, were also associated with changes in phenotypic aging. Notably, some associations differed by sex, highlighting potential sex-specific patterns in the relationship between lifestyle factors and biological aging.

## Discussion

By focusing on within-individual changes in PhenoAge, the influence of time-invariant baseline differences (such as genetic makeup) is substantially reduced. In line with this aim, SNP heritability estimated using “Genome-wide Complex Trait Analysis” (GCTA 1.93.2beta) (Yang et al., 2011) was 14.03% (SE = 1.49%) for baseline PhenoAge (Lin, 2022), but only 0.41% (SE = 0.47%) for △PhenoAge. This suggests that changes in PhenoAge are less influenced by common genetic variants and may more sensitively reflect environmental and lifestyle factors.

Our previous study using TWB DNA methylation data showed that abdominal obesity and general obesity were significantly associated with epigenetic age acceleration in men and women, respectively (Lin et al., 2021). Consistent with this epigenetic evidence, the present phenotypic analyses indicated that △WC (an index of abdominal obesity) and △BMI (an index of general obesity) were associated with △PhenoAge in men and women, respectively. The known differences in fat distribution may help explain these sex-specific associations. In particular, men tend to accumulate more visceral adipose tissue, whereas women generally develop more subcutaneous fat (Kim et al., 2025).

Living alone was associated with an acceleration of PhenoAge in men (β = 0.4032 years, SE = 0.0884, p < 0.001), but this association was not found in women. This sex-specific pattern can reflect previous studies reporting adverse health outcomes related to living alone, mostly in men. For example, a large German cohort study including 3,596 men and 3,420 women found that living alone was associated with increased mortality risk in men but not in women (Kandler et al., 2007). In addition, a systematic review and meta-analysis reported that men living alone were more likely to be frail, whereas no such association was observed among women (Kojima et al., 2020).

Diet2 (When you eat fish or meat, do you prefer to cook it with oil, such as by frying? 1: Always; …; 5: Never), Diet5 (When you eat soy foods, do you prefer them deep-fried, such as fried tofu? 1: Always; …; 5: Never), Diet16 (Do you eat at least two kinds of vegetables a day? 1: Always; …; 5: Never), and Diet17 (Do you intentionally eat less when having meat? 1: Always; …; 5: Never) were selected as significant predictors for men’s and women’s △PhenoAge models. These four dietary questions emphasized the importance of reducing consumption of fried foods and meat, and of eating a variety of vegetables.

Tea consumption was associated with a 0.1053-year (SE = 0.0476, p < 0.05) decrease in men’s PhenoAge. Routinely taking vitamins and mineral supplements was associated with a faster PhenoAge acceleration in women (p < 0.01). Supplements do not necessarily provide benefits for healthy people with a balanced diet. Multiple studies have found that taking additional vitamins and minerals does not improve overall health for the general population (Zhang et al., 2020). Even more concerning, dietary supplements may increase the risk of cancer (Jabbari et al., 2024). Eating supper within an hour of bedtime was associated with a 0.0700-year (SE = 0.0307) increase in women’s PhenoAge (p < 0.05). Possible underlying mechanisms include metabolic disruption and cardio-renal risk due to mis-timed meals (Benjamin et al., 2024; Davis et al., 2022).

Exposure to incense burning was associated with a faster rate of aging in both men and women (p < 0.001). A 0.2747-year (SE = 0.0524) and 0.1633-year (SE = 0.0322) increase in PhenoAge acceleration was observed in men and women, respectively. This result may reflect the harmful effects of pollutants released during incense burning. As reported by prior studies, incense exposure is associated with accelerated cognitive aging and intellectual decline (Wong et al., 2020).

Employment stability is associated with better mental and physical health arising from a sense of achievement, increased physical activity, and sustained social connections (Drake and Wallach, 2020). “Staying at the same job throughout the career” was associated with a 0.3710-year (SE = 0.0758; p < 0.001) and 0.0981-year (SE = 0.0500; p < 0.05) decrease in △PhenoAge for men and women, respectively. Employment stability may help develop long-lasting workplace relationships. In Taiwanese society, traditional gender roles often position men as primary economic providers (Iida, 2023), a pattern that may be associated with greater employment continuity. This is consistent with the stronger association observed in men.

Because sport habits were assessed at both baseline and follow-up, the association between changes in sport habits and △PhenoAge was examined using partial correlation analysis. Changes in sport habits were categorized as improvement (SPONoYes), sustained participation (SPOYesYes), sustained non-participation (SPONoNo), and deterioration (SPOYesNo). The partial correlation coefficients showed an increasing trend in △PhenoAge from improvement to deterioration, with SPOYesNo (deterioration in sport habits) being positively correlated with △PhenoAge (p < 0.05). Although these sport habit variables were not selected as predictors in the best-subset analysis, the observed pattern of partial correlations suggests that maintaining regular physical activity may help slow the aging process.

The observed associations between lifestyle factors and ΔPhenoAge may reflect underlying biological mechanisms. Several of these factors, such as obesity, unhealthy dietary habits, and physical inactivity, are known to influence systemic inflammation, oxidative stress, and metabolic regulation, all of which are closely linked to biological aging processes (Ellulu et al., 2017; Li et al., 2023; Militello et al., 2024). For example, excess adiposity is associated with chronic low-grade inflammation (Khanna et al., 2022). In addition, unhealthy dietary patterns and physical inactivity may contribute to metabolic dysregulation and impaired cellular repair mechanisms (Huston, 2022).

Furthermore, PhenoAge has been associated with multiple age-related outcomes, including cardiovascular disease, frailty, and mortality (Gao et al., 2025; Kwan et al., 2025; Xu and Xu, 2024). Therefore, the lifestyle factors identified in this study may have broader implications for the development of age-related pathologies. Although causal inference cannot be claimed in the present study, these findings suggest that modifiable lifestyle factors may influence biological aging trajectories and contribute to healthy aging.

To better understand the biological relevance of lifestyle factors, partial correlations between lifestyle variables and individual components of PhenoAge were examined while adjusting for chronological age (Supplementary Figure S2). Adiposity-related measures (BMI, body fat percentage, and waist circumference) showed consistent correlations with multiple biomarkers, including fasting glucose, white blood cell count, and creatinine. The observed patterns, particularly higher glucose and white blood cell count, are consistent with metabolic dysregulation and chronic low-grade inflammation, as suggested by previous studies (Khanna et al., 2022; LeRoith, 2007).

In addition, individuals who reported consistent physical activity at both time points (SPOYesYes) tended to have lower fasting glucose and white blood cell counts, whereas those who were inactive at both time points (SPONoNo) showed higher levels of these biomarkers. These findings suggest that sustained physical activity is associated with more favorable metabolic and inflammatory profiles, in line with previous evidence (Magni et al., 2025). In contrast, dietary variables showed weaker, less consistent correlations with individual biomarkers, which may reflect differences in questionnaire design, scoring direction, and the inherent limitations of self-reported dietary variables assessed on a 5-point scale. Taken together, these findings suggest that metabolic and inflammatory pathways may play a central role in linking lifestyle factors to phenotypic aging.

This study has several limitations. First, although within-individual changes in PhenoAge were examined, residual confounding from unmeasured factors may remain. Therefore, the findings should be interpreted as associations rather than causal relationships. Second, lifestyle factors were primarily self-reported and may be subject to measurement error or reporting bias. Finally, the study population was drawn from the TWB. The generalizability of these findings to other populations with different genetic backgrounds or lifestyle patterns may be limited.

To sum up, a healthy lifestyle was associated with lower PhenoAge. Men and women exhibited distinct lifestyle factors associated with aging. Obesity was strongly associated with higher PhenoAge. Cooking by oneself showed opposite effects on PhenoAge changes between men and women: it was associated with acceleration in men but deceleration in women. Exposure to incense burning was a risk factor for aging in both sexes.

## Data Availability

The original contributions presented in the study are included in the article/Supplementary Material, further inquiries can be directed to the corresponding author.

## References

[B1] Ahadi S. Zhou W. Schussler-Fiorenza Rose S. M. Sailani M. R. Contrepois K. Avina M. (2020). Personal aging markers and ageotypes revealed by deep longitudinal profiling. Nat. Med. Jan. 26, 83–90. 10.1038/s41591-019-0719-5 PMC7301912 31932806

[B2] Benjamin J. I. Pati P. Luong T. Liu X. De Miguel C. Pollock J. S. (2024). Chronic mistimed feeding results in renal fibrosis and disrupted circadian blood pressure rhythms. Am. J. Physiol. Ren. Physiol. 327 (327), F683–F696. 10.1152/ajprenal.00047.2024 39205662 PMC11563648

[B3] Davis R. Rogers M. Coates A. M. Leung G. K. W. Bonham M. P. (2022). The impact of meal timing on risk of weight gain and development of obesity: a review of the current evidence and opportunities for dietary intervention. Curr. Diab Rep. 22, 147–155. 10.1007/s11892-022-01457-0 35403984 PMC9010393

[B4] Drake R. E. Wallach M. A. (2020). Employment is a critical mental health intervention. Epidemiol. Psych. Sci. 29, e178. 10.1017/S2045796020000906 33148366 PMC7681163

[B5] Ellulu M. S. Patimah I. Khaza'ai H. Rahmat A. Abed Y. (2017). Obesity and inflammation: the linking mechanism and the complications. Arch. Med. Sci. Jun 13, 851–863. 10.5114/aoms.2016.58928 PMC5507106 28721154

[B6] Feng Y. A. Chen C. Y. Chen T. T. Kuo P. H. Hsu Y. H. Yang H. I. (2022). Taiwan biobank: a rich biomedical research database of the Taiwanese population. Cell Genom 2, 100197. 10.1016/j.xgen.2022.100197 36776991 PMC9903657

[B7] Feraco A. Armani A. Amoah I. Guseva E. Camajani E. Gorini S. (2024). Assessing gender differences in food preferences and physical activity: a population-based survey. Front. Nutr. 11, 1348456. 10.3389/fnut.2024.1348456 38445208 PMC10912473

[B8] Gao Y. J. Gao K. Shi R. J. Huang X. R. Dang P. Z. Liu H. (2025). Association between phenotypic age and in-hospital outcomes in patients with acute myocardial infarction: a retrospective observational study. Ijc Heart Vasc. Jun 58, 101670. 10.1016/j.ijcha.2025.101670 40235940 PMC11997336

[B9] Huston P. (2022). A sedentary and unhealthy lifestyle fuels chronic disease progression by changing interstitial cell behaviour: a network analysis. Front. Physiol. 13, 904107. 10.3389/fphys.2022.904107 35874511 PMC9304814

[B10] Iida A. (2023). How do traditional gender roles influence women's lives in Taiwan? An investigation of highly educated women's willingness to create families. E Netherl. Mar. 40, 81–100. 10.1007/s12140-022-09392-3

[B11] Jabbari P. Yazdanpanah O. Benjamin D. J. Rezazadeh Kalebasty A. (2024). Supplement use and increased risks of cancer: unveiling the other side of the coin. Cancers (Basel) 16, 880. 10.3390/cancers16050880 38473246 PMC10930792

[B12] Kandler U. Meisinger C. Baumert J. Löwel H. Grp K. S. (2007). Living alone is a risk factor for mortality in men but not women from the general population:: a prospective cohort study. Bmc Public Health 7, 335. 10.1186/1471-2458-7-335 18336722 PMC2225416

[B13] Khanna D. Khanna S. Khanna P. Kahar P. Patel B. M. (2022). Obesity: a chronic low-grade inflammation and its markers. Cureus 14, e22711. 10.7759/cureus.22711 35386146 PMC8967417

[B14] Kim S. (2015). Ppcor: an R package for a fast calculation to semi-partial correlation coefficients. Commun. Stat. Appl. Methods 22, 665–674. 10.5351/CSAM.2015.22.6.665 26688802 PMC4681537

[B15] Kim H. Kim S. E. Sung M. K. (2025). Sex and gender differences in obesity: biological, sociocultural, and clinical perspectives. World J. Mens. Health 43, 758–772. 10.5534/wjmh.250126 40676890 PMC12505483

[B16] Klemera P. Doubal S. (2006). A new approach to the concept and computation of biological age. Mech. Ageing Dev. Mar. 127, 240–248. 10.1016/j.mad.2005.10.004 16318865

[B17] Kojima G. Taniguchi Y. Kitamura A. Fujiwara Y. (2020). Is living alone a risk factor of frailty? A systematic review and meta-analysis. Ageing Res. Rev. 59, 101048. 10.1016/j.arr.2020.101048 32173535

[B18] Kuo C. L. Pilling L. C. Liu Z. Atkins J. L. Levine M. E. (2021). Genetic associations for two biological age measures point to distinct aging phenotypes. Aging Cell 20, e13376. 10.1111/acel.13376 34038024 PMC8208797

[B19] Kwan K. J. S. Xie S. S. Li H. L. Lin X. G. Lu Y. J. Chen B. (2025). Evaluating the potential of phenotypic age to enhance cardiovascular risk prediction over chronological age in the UK biobank. Sci. Rep. 15 (15), 27858. 10.1038/s41598-025-12495-5 40738943 PMC12310943

[B20] LeRoith D. (2007). Dyslipidemia and glucose dysregulation in overweight and obese patients. Clin. Cornerstone 8, 38–52. 10.1016/s1098-3597(07)80027-5 18452841

[B21] Levine M. E. (2013). Modeling the rate of senescence: can estimated biological age predict mortality more accurately than chronological age? J. Gerontol. a-Biol 68, 667–674. 10.1093/gerona/gls233 23213031 PMC3660119

[B22] Levine M. E. Lu A. T. Quach A. Chen B. H. Assimes T. L. Bandinelli S. (2018). An epigenetic biomarker of aging for lifespan and healthspan. Aging (Albany NY) 10, 573–591. 10.18632/aging.101414 29676998 PMC5940111

[B23] Li B. Tang X. Le G. (2023). Dietary habits and metabolic health. Nutrients 15, 3975. 10.3390/nu15183975 37764759 PMC10536179

[B24] Lin W. Y. (2022). Lifestyle factors and genetic variants on 2 biological age measures: evidence from 94 443 Taiwan biobank participants. J. Gerontol. A Biol. Sci. Med. Sci. 77, 1189–1198. 10.1093/gerona/glab251 34427645

[B25] Lin W. Y. Wang Y. C. Teng I. H. Liu C. Lou X. Y. (2021). Associations of five obesity metrics with epigenetic age acceleration: evidence from 2,474 Taiwan biobank participants. Obes. (Silver Spring) 29, 1731–1738. 10.1002/oby.23255 34472716

[B26] Liu Z. Y. Kuo P. L. Horvath S. Crimmins E. Ferrucci L. Levine M. (2019). A new aging measure captures morbidity and mortality risk across diverse subpopulations from NHANES IV: a cohort study (vol 15, e1002718, 2018). Plos Med. 16, e1002760. 10.1371/journal.pmed.1002718 30596641 PMC6312200

[B27] Lloyd-Jones D. M. Hong Y. Labarthe D. Mozaffarian D. Appel L. J. Van Horn L. (2010). Defining and setting national goals for cardiovascular health promotion and disease reduction: the American heart Association's strategic impact goal through 2020 and beyond. Circulation 121, 586–613. 10.1161/CIRCULATIONAHA.109.192703 20089546

[B28] Magni O. Arnaoutis G. Panagiotakos D. (2025). The impact of exercise on chronic systemic inflammation: a systematic review and meta-meta-analysis. Sport Sci. hlth. 21, 1405–1417. 10.1007/s11332-025-01445-3

[B29] Militello R. Luti S. Gamberi T. Pellegrino A. Modesti A. Modesti P. A. (2024). Physical activity and oxidative stress in aging. Antioxidants (Basel) 13, 557. 10.3390/antiox13050557 38790662 PMC11117672

[B30] Rutledge J. Oh H. Wyss-Coray T. (2022). Measuring biological age using omics data. Nat. Rev. Genet. 23, 715–727. 10.1038/s41576-022-00511-7 35715611 PMC10048602

[B31] Wei C. Y. Yang J. H. Yeh E. C. Tsai M. F. Kao H. J. Lo C. Z. (2021). Genetic profiles of 103,106 individuals in the Taiwan biobank provide insights into the health and history of han Chinese. Npj Genom Med. 6: 10. 10.1038/s41525-021-00178-9 33574314 PMC7878858

[B32] Wong A. Lou W. Ho K. F. Yiu B. K. Lin S. Chu W. C. (2020). Indoor incense burning impacts cognitive functions and brain functional connectivity in community older adults. Sci. Rep. 10, 7090. 10.1038/s41598-020-63568-6 32341386 PMC7184605

[B33] Xu X. Xu Z. (2024). Association between phenotypic age and the risk of mortality in patients with heart failure: a retrospective cohort study. Clin. Cardiol. 47, e24321. 10.1002/clc.24321 39114957 PMC11307102

[B34] Yang J. Lee S. H. Goddard M. E. Visscher P. M. (2011). GCTA: a tool for genome-wide complex trait analysis. Am. J. Hum. Genet. 88, 76–82. 10.1016/j.ajhg.2010.11.011 21167468 PMC3014363

[B35] Zhang W. G. Bai X. J. Sun X. F. Cai G. Y. Bai X. Y. Zhu S. Y. (2014). Construction of an integral formula of biological age for a healthy Chinese population using principle component analysis. J. Nutr. Health Aging 18, 137–142. 10.1007/s12603-013-0345-8 24522464 PMC12879004

[B36] Zhang F. F. Barr S. I. McNulty H. Li D. Blumberg J. B. (2020). Health effects of vitamin and mineral supplements. BMJ. 369, m2511. 10.1136/bmj.m2511 32601065 PMC7322674

