# Supplementary material for: Modifiable lifestyle factors and 4.9-Year changes in phenotypic age in the Taiwan biobank
Source: Front Aging. 2026 May 7;7:1802176. doi: 10.3389/fragi.2026.1802176 (PMC13189777; doi:10.3389/fragi.2026.1802176)
Supplement: Supplementary file 1 [file DataSheet1.pdf]

Supplementary Materials of

Modifiable Lifestyle Factors and 4.9-Year Changes in Phenotypic Age in the Taiwan Biobank

Wan-Yu Lin [1,2*]

[1] Institute of Health Data Analytics and Statistics, College of Public Health, National Taiwan University, Taipei, Taiwan
[2] Master of Public Health Program, College of Public Health, National Taiwan University, Taipei, Taiwan

*** Correspondence:**
Corresponding Author: Wan-Yu Lin, Ph.D. (https://orcid.org/0000-0002-3385-4702)
E-mail: linwy@ntu.edu.tw
Room 501, No. 17, Xu-Zhou Road, Taipei 100, Taiwan
Phone/Fax: +886-2-33668106

Contents

Table S1. The 17 diet-related questions in the Taiwan Biobank questionnaire.. 2
Figure S1. The changes in phenotypic age and chronological age for 200 randomly sampled men and women . 4
Figure S2. Correlations between lifestyle factors and individual components of PhenoAge in men and women 6
References:.. 7

Table S1. The 17 diet-related questions in the Taiwan Biobank questionnaire

Dietary Habits/ Food Category	Always	Most of the time	Half of the time	Seldom	Never
1. When you eat meat (such as pork, beef, mutton, chicken, duck, goose, etc.), do you eat it with fat, suet, or skin?	1	2	3	4	5
2. When you eat fish or meat, do you prefer to cook it with oil (such as by frying, deep-frying, braising, or steaming fish and topping it with oil)?	1	2	3	4	5
3. When you eat vegetables, do you prefer cooking them in a stir-fry way?	1	2	3	4	5
4. When you eat rice or noodles (staple food), do you eat them with marinade, gravy, or lard?	1	2	3	4	5
5. When you eat soy foods, do you prefer them deep-fried (such as fried tofu, stinky tofu, or tofu skin)?	1	2	3	4	5
6. When you eat bread, do you spread butter, plant-based butter (margarine), or mayonnaise?	1	2	3	4	5
7. When you have meals, do you add additional salt, soy sauce, chili sauce, or any other seasoning?	1	2	3	4	5
8. Are you used to having pickles, fermented tofu, and fermented soybeans as side dishes in a meal?	1	2	3	4	5
9. Do you eat fruits or vegetables instead of high-fat snacks (such as chips, cakes, doughnuts, etc.) when you have snacks?	1	2	3	4	5
10. When you prepare meat (such as pork, beef, mutton, chicken, duck, goose, etc.) for a meal, do you cook it roasted or braised instead of deep-frying?	1	2	3	4	5
11. If a food product has a low-fat option (such as low-fat ice cream, low-fat milk, skim milk, low-fat salad sauce, etc.), would you choose it instead of the regular product?	1	2	3	4	5
12. Do you eat food with low-sodium ingredients (such as low-sodium salt, lower-sodium soy sauce, etc.)?	1	2	3	4	5
13. Would you like to eat lower-fat meat (such as fish or chicken) instead of higher-fat meat (such as beef or pork)?	1	2	3	4	5
14. Would you choose to eat lean meat instead of fatty meat?	1	2	3	4	5
15. Would you choose a vegetarian and light	1	2	3	4	5

Dietary Habits/ Food Category	Always	Most of the time	Half of the time	Seldom	Never
diet in certain meals to reduce the intake of higher-fat foods such as meat or fat?					
16. Do you eat at least two kinds of vegetables a day?	1	2	3	4	5
17. Do you intentionally eat less when having meat?	1	2	3	4	5

This table was Supplementary Table S4 in Lo and Lin (Lo and Lin 2022).

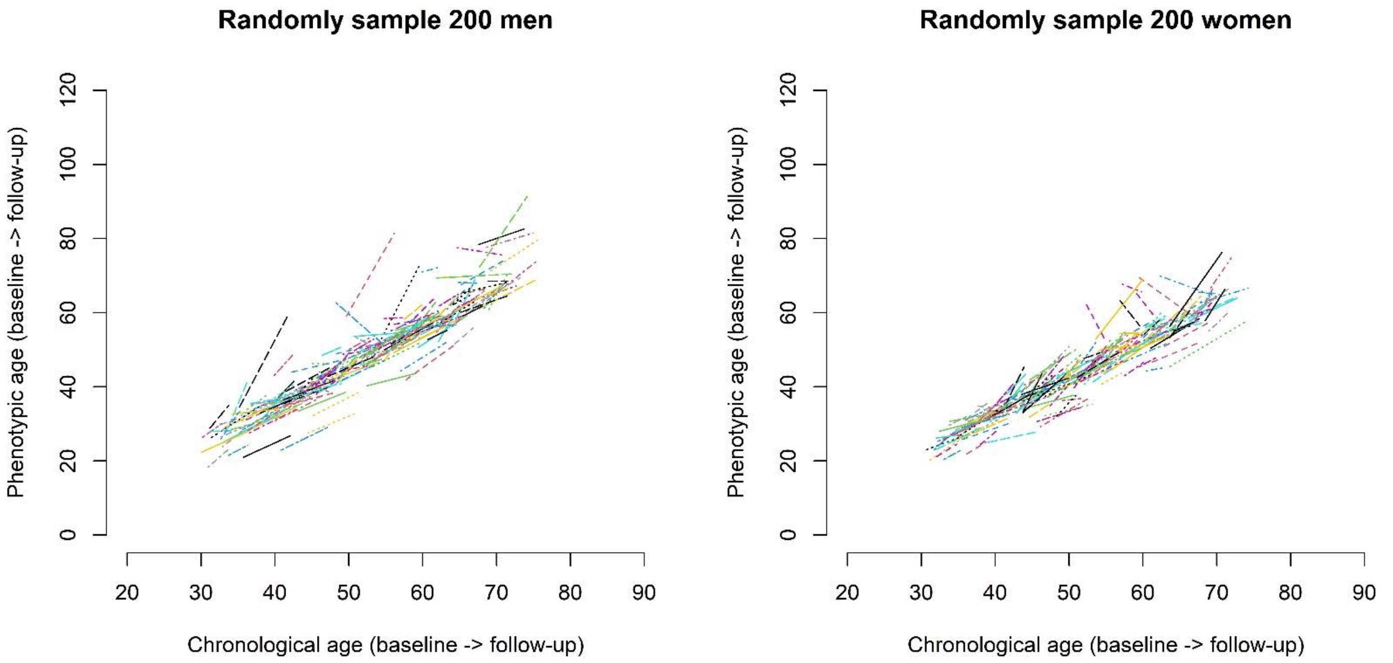

Figure S1. The changes in phenotypic age and chronological age for 200 randomly sampled men and women

The *x*-axis shows chronological age, and the y-axis depicts phenotypic age. Chronological age (in years) was calculated as the difference between the survey date (when urine and blood samples, and lifestyle data were collected) and the date of birth, divided by 365.25; decimal values were retained.

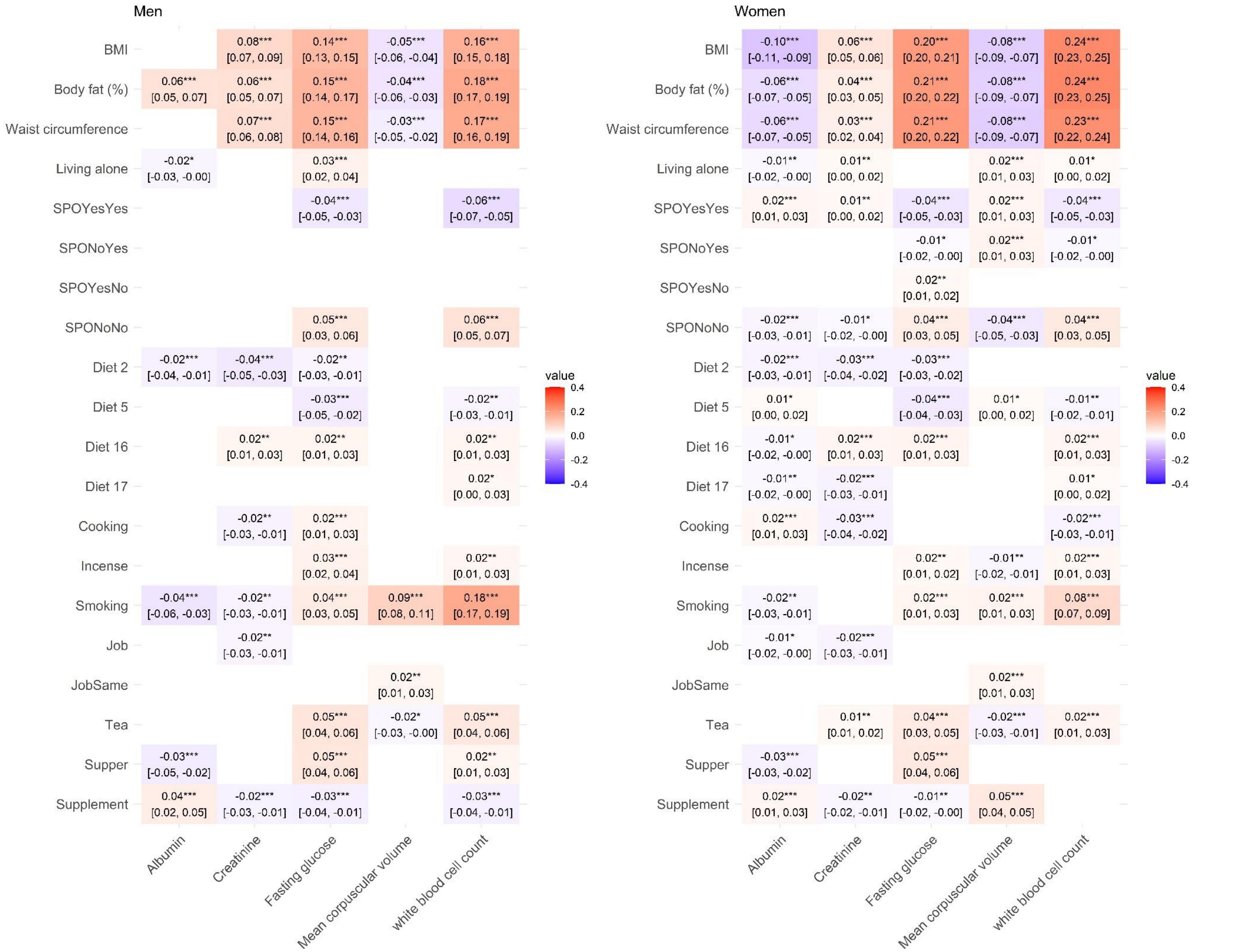

Men
BMI
0.08*** [0.07, 0.09]
0.14*** [0.13, 0.15]
-0.05*** [-0.06, -0.04]
0.16*** [0.15, 0.18]
Body fat (%)
0.06*** [0.05, 0.07]
0.06*** [0.05, 0.07]
0.15*** [0.14, 0.17]
-0.04*** [-0.06, -0.03]
0.18*** [0.17, 0.19]
Waist circumference
0.07*** [0.06, 0.08]
0.15*** [0.14, 0.16]
-0.03*** [-0.05, -0.02]
0.17*** [0.16, 0.19]
Living alone
-0.02* [-0.03, -0.00]
0.03*** [0.02, 0.04]
SPOYesYes
-0.04*** [-0.05, -0.03]
-0.06*** [-0.07, -0.05]
SPONoYes
SPOYesNo
SPONoNo
0.05*** [0.03, 0.06]
0.06*** [0.05, 0.07]
Diet 2
-0.02*** [-0.04, -0.01]
-0.04*** [-0.05, -0.03]
-0.02** [-0.03, -0.01]
Diet 5
-0.03*** [-0.05, -0.02]
-0.02** [-0.03, -0.01]
Diet 16
0.02** [0.01, 0.03]
0.02** [0.01, 0.03]
0.02** [0.01, 0.03]
Diet 17
0.02* [0.00, 0.03]
Cooking
-0.02** [-0.03, -0.01]
0.02*** [0.01, 0.03]
Incense
0.03*** [0.02, 0.04]
0.02** [0.01, 0.03]
Smoking
-0.04*** [-0.06, -0.03]
-0.02** [-0.03, -0.01]
0.04*** [0.03, 0.05]
0.09*** [0.08, 0.11]
0.18*** [0.17, 0.19]
Job
-0.02** [-0.03, -0.01]
JobSame
0.02** [0.01, 0.03]
Tea
0.05*** [0.04, 0.06]
-0.02* [-0.03, -0.00]
0.05*** [0.04, 0.06]
Supper
-0.03*** [-0.05, -0.02]
0.05*** [0.04, 0.06]
0.02** [0.01, 0.03]
Supplement
0.04*** [0.02, 0.05]
-0.02*** [-0.03, -0.01]
-0.03*** [-0.04, -0.01]
-0.03*** [-0.04, -0.01]
Albumin
Creatinine
Fasting glucose
Mean corpuscular volume
white blood cell count
value
0.4
0.2
0.0
-0.2
-0.4
Women
BMI
-0.10*** [-0.11, -0.09]
0.06*** [0.05, 0.06]
0.20*** [0.20, 0.21]
-0.08*** [-0.09, -0.07]
0.24*** [0.23, 0.25]
Body fat (%)
-0.06*** [-0.07, -0.05]
0.04*** [0.03, 0.05]
0.21*** [0.20, 0.22]
-0.08*** [-0.09, -0.07]
0.24*** [0.23, 0.25]
Waist circumference
-0.06*** [-0.07, -0.05]
0.03*** [0.02, 0.04]
0.21*** [0.20, 0.22]
-0.08*** [-0.09, -0.07]
0.23*** [0.22, 0.24]
Living alone
-0.01** [-0.02, -0.00]
0.01** [0.00, 0.02]
0.02*** [0.01, 0.03]
0.01* [0.00, 0.02]
SPOYesYes
0.02*** [0.01, 0.03]
0.01** [0.00, 0.02]
-0.04*** [-0.05, -0.03]
0.02*** [0.01, 0.03]
-0.04*** [-0.05, -0.03]
SPONoYes
-0.01* [-0.02, -0.00]
0.02*** [0.01, 0.03]
-0.01* [-0.02, -0.00]
SPOYesNo
0.02** [0.01, 0.02]
SPONoNo
-0.02*** [-0.03, -0.01]
-0.01* [-0.02, -0.00]
0.04*** [0.03, 0.05]
-0.04*** [-0.05, -0.03]
0.04*** [0.03, 0.05]
Diet 2
-0.02*** [-0.03, -0.01]
-0.03*** [-0.04, -0.02]
-0.03*** [-0.03, -0.02]
Diet 5
0.01* [0.00, 0.02]
-0.04*** [-0.04, -0.03]
0.01* [0.00, 0.02]
-0.01** [-0.02, -0.01]
Diet 16
-0.01* [-0.02, -0.00]
0.02*** [0.01, 0.03]
0.02*** [0.01, 0.03]
0.02*** [0.01, 0.03]
Diet 17
-0.01** [-0.02, -0.00]
-0.02*** [-0.03, -0.01]
0.01* [0.00, 0.02]
Cooking
0.02*** [0.01, 0.03]
-0.03*** [-0.04, -0.02]
-0.02*** [-0.03, -0.01]
Incense
0.02** [0.01, 0.02]
-0.01** [-0.02, -0.01]
0.02*** [0.01, 0.03]
Smoking
-0.02** [-0.03, -0.01]
0.02*** [0.01, 0.03]
0.02*** [0.01, 0.03]
0.08*** [0.07, 0.09]
Job
-0.01* [-0.02, -0.00]
-0.02*** [-0.03, -0.01]
JobSame
0.02*** [0.01, 0.03]
Tea
0.01** [0.01, 0.02]
0.04*** [0.03, 0.05]
-0.02*** [-0.03, -0.01]
0.02*** [0.01, 0.03]
Supper
-0.03*** [-0.03, -0.02]
0.05*** [0.04, 0.06]
Supplement
0.02*** [0.01, 0.03]
-0.02** [-0.02, -0.01]
-0.01** [-0.02, -0.00]
0.05*** [0.04, 0.05]
Albumin
Creatinine
Fasting glucose
Mean corpuscular volume
white blood cell count
value
0.4
0.2
0.0
-0.2
-0.4

Figure S2. Correlations between lifestyle factors and individual components of PhenoAge in men and women

Heatmaps show partial correlation coefficients between lifestyle factors and individual components of PhenoAge at follow-up (adjusted for chronological age at follow-up), stratified by sex. The PhenoAge component biomarkers include albumin, creatinine, fasting glucose, mean corpuscular volume, and white blood cell count. Values represent correlation coefficients, with corresponding 95% confidence intervals shown in brackets. Statistical significance is marked with *, **, and ***, representing a Benjamini-Hochberg false discovery rate (FDR) (Benjamini and Hochberg 1995) less than 0.05, 0.01, and 0.001, respectively.

BMI: Body mass index at follow-up.
Body fat (%): Body fat percentage at follow-up.
Waist circumference: Waist circumference at follow-up.
Living alone: Living alone at follow-up (1 = yes, 0 = no).
SPO categories (regular exercise defined as ≥ 30 min per session, ≥ 3 times per week):
- SPOYesYes: Regular exercise at both baseline and follow-up.
- SPONoYes: Regular exercise at follow-up but not at baseline.
- SPOYesNo: Regular exercise at baseline but not at follow-up.
- SPONoNo: No regular exercise at either baseline or follow-up.

Diet2: When you eat fish or meat, do you prefer to cook it with oil (such as by frying, deep-frying, braising, or steaming fish and topping it with oil)? 1: Always; 2: Most of the time; 3: Half of the time; 4: Seldom; 5: Never.
Diet5: When you eat soy foods, do you prefer them deep-fried (such as fried tofu, stinky tofu, or tofu skin)? 1: Always; 2: Most of the time; 3: Half of the time; 4: Seldom; 5: Never.
Diet16: Do you eat at least two kinds of vegetables a day? 1: Always; 2: Most of the time; 3: Half of the time; 4: Seldom; 5: Never.
Diet17: Do you intentionally eat less when having meat? 1: Always; 2: Most of the time; 3: Half of the time; 4: Seldom; 5: Never.
Cooking = 1: have not cooked meals by yourselves for more than six months; Cooking = 2: cooked meals by yourselves sometimes; Cooking = 3: have cooked by yourselves for over six months.
Incense: being exposed to incense burning (e.g., during worship or the use of incense powder or rings), mosquito coils (traditional, liquid electric, or electric mosquito repellent), or fragrances (such as essential oils, aromatherapy, air fresheners, sprays, or scented candles) for at least five minutes in the past year before joining the TWB. (1 = yes, 0 = no).
Smoking: having smoked cigarettes for at least 6 months when joining the TWB. (1 = yes, 0 = no).
Job: Currently having a job. (1 = yes, 0 = no).
JobSame: Stay at the same job throughout the career. (1 = yes, 0 = no).
Tea: consuming tea (containing tea leaves, excluding herbal tea) at least once daily within six months before joining the TWB. (1 = yes, 0 = no).
Supper: eating supper within an hour before bedtime (including milk and wine). (1 = yes, 0 = no).
Supplement = 1: do not take vitamins, minerals, or supplements in the past month before joining the TWB; Supplement = 2: sometimes (not regularly) take vitamins, minerals, or supplements in the past month before joining the TWB; Supplement = 3: regularly take vitamins, minerals, or supplements in the past month before joining the TWB.

References:

Benjamini Y, Hochberg Y. 1995. Controlling the false discovery rate: A practical and powerful approach to multiple testing. J R Stat Soc B.57:289-300.

Lo YH, Lin WY. 2022. Cardiovascular health and four epigenetic clocks. Clin Epigenetics. Jun 9;14:73. Epub 20220609.
